# Thrombocytopenia and venous thromboembolic events after BNT162b2, CoronaVac, ChAdOx1 vaccines and SARS-CoV-2 infection: a self-controlled case series study

**DOI:** 10.1038/s41598-023-47486-x

**Published:** 2023-11-22

**Authors:** Norazida Ab Rahman, Ming Tsuey Lim, Fei Yee Lee, Wee Kee Wo, Hee Sheong Yeoh, Kalaiarasu M. Peariasamy, Sheamini Sivasampu, the SAFECOVAC Study Group, Azuana Ramli, Sing Chet Lee, Sim Mei Choo, Maheshwara Rao Appanan, Teck Long King, Chia How Yen, Emelyne Bani Anak Jam, Fatihah Mahmud, Fariz Safhan Mohamad Nor, Muhammad Hazrul Badrul Hisham, Siti Nurhafizah Saharudin, Nor Aliya Ayub, Raj Kumar Sevalingam, Rashidah Bahari, Nor Nadziroh Ibrahim, Nurain Mohd Noor, Lisa Mohamed Nor, Nurul Huda Zainal Abidin, Hin Seng Wong, Siti Nur Akmal Ghazali, Nurul Afifah Rozkhaidi, Norzubaidatulhikmah Shaduqi, Hanisah Hossain, Jeannette Jieni Lay, Nor Azizah Mohamad Nazri

**Affiliations:** 1 Institute for Clinical Research, National Institutes of Health, Block B4, No 1, Jalan Setia Murni U13/52, 40170 Shah Alam, Selangor Malaysia; 2 Clinical Research Centre, Selayang Hospital, Ministry of Health, Kepong, Selangor Malaysia; 3 National Pharmaceutical Regulatory Agency, Ministry of Health, Shah Alam, Selangor Malaysia; 4 National Pharmaceutical Regulatory Agency, Petaling Jaya, Selangor Malaysia; 5 Disease Control Division, Ministry of Health, Putrajaya, Malaysia; 6 Clinical Research Centre, Sarawak General Hospital, Kuching, Sarawak Malaysia; 7 Clinical Research Centre, Queen Elizabeth II Hospital, Kota Kinabalu, Sabah Malaysia; 8 Clinical Research Centre, Tengku Ampuan Afzan Hospital, Kuantan, Pahang Malaysia; 9 Clinical Research Centre, Shah Alam Hospital, Shah Alam, Selangor Malaysia; 10 Clinical Research Centre, Kuala Lumpur Hospital, Kuala Lumpur, Malaysia; 11 Clinical Research Centre, Putrajaya Hospital, Putrajaya, Malaysia; 12 Clinical Research Centre, Selayang Hospital, Kepong, Selangor Malaysia; 13 Institute for Clinical Research, National Institutes of Health, Shah Alam, Selangor Malaysia

**Keywords:** Epidemiology, Outcomes research

## Abstract

This study assessed the association between COVID-19 vaccines, SARS-CoV-2 infection and the risk of thrombocytopenia and venous thromboembolism (VTE). This self-controlled case series study used hospital records between 1st February 2021 and 28th February 2022 linked to the national immunisation registry and COVID-19 surveillance data in Malaysia. Conditional Poisson regression was used to estimate incidence rate ratios (IRR) of events in the risk period (day 1–21 post-exposure) relative to control period with the corresponding 95% confidence interval (CI) adjusted for calendar period. We found no significant increased risk of thrombocytopenia in 1–21 days following BNT162b2, CoronaVac and ChAdOx1 vaccines while the risk was increased following SARS-CoV-2 infection (IRR 15.52, 95% CI 13.38–18.00). Similarly, vaccination with BNT162b2, CoronaVac, or ChAdOx1 was not associated with an increased risk of VTE during the 1–21 days risk period. SARS-CoV-2 infection was associated with increased risk of VTE (IRR 39.84, 95% CI 27.45–32.44). Our findings showed low event rates of thrombocytopenia and VTE following booster vaccination with comparable safety profiles between those who received homologous and heterologous booster combinations. Our findings showed the risk of thrombocytopenia and VTE was not increased after COVID-19 vaccination while the risks were substantially higher after SARS-CoV-2 infection.

## Introduction

Since the global deployment of COVID-19 vaccines, billions of doses have been administered to the population worldwide to combat the COVID-19 pandemic. Whilst COVID-19 vaccines authorised for use have been shown to be safe and effective^1–3^, these vaccines continue to be monitored in the post-marketing phase to gather data on safety profile. Two of the most serious side effects, thrombocytopenia and thrombosis, were first reported to regulatory authorities in the first quarter of 2021 following rare cases that occurred shortly after COVID-19 vaccination^4,5^. Cases were mostly linked to the adenoviral vector-based vaccines, ChAdOx1 nCoV-19 (AstraZeneca) and Ad26.COV2.S (Janssen), where the vaccine appear to stimulate autoantibodies to platelet factor 4 (PF4), resulting in platelet activation^6–9^. The condition also referred to as thrombosis with thrombocytopenia syndrome, appeared to be a rare side effect of the vector-based COVID-19 vaccine^4,10,11^. This resulted in several countries pausing or limiting the use of ChAdOx1 and Ad26.COV2-S vaccine.

Cases of thrombosis or thrombocytopenia following administration of mRNA-based vaccine were similarly observed, but at a smaller extent compared to ChAdOx1 with less conclusive findings^12–14^. Data concerning the association of these events with inactivated COVID-19 vaccine remains scarce, with limited studies that described the risk for CoronaVac. Of note, different vaccine platforms have variations in their safety profiles since the mechanisms to trigger an immune response are different. Population-based studies thus far predominantly from Western countries that described the risk of events in the population with most comparisons between mRNA and adenoviral vector COVID-19 vaccine. Limited evidence is available on whether such risks are similarly observed in Asian populations, given that differences exist in the genetic and risk factors between Western and Asian populations that may potentially affect the vaccine safety profile^15,16^. Furthermore, evidence suggests that SARS-CoV-2 infection is a risk factor for thrombosis and thrombocytopenia^12,17–19^. Therefore, a comparison of the risk of these events after SARS-CoV-2 vaccination and SARS-CoV-2 infection will provide context for risk assessment.

In Malaysia, the national immunisation program against COVID-19 commenced in February 2021 and since then, more than 70 million doses of COVID-19 vaccines have been administered with over 80% of the adult population being fully vaccinated as of June 2022^20^. Malaysia primarily used the mRNA vaccine BNT162b2 (Pfizer BioNTech), inactivated vaccine CoronaVac (Sinovac), and adenoviral vector vaccine ChAdOx1 (AstraZeneca), while other vaccines approved for use in Malaysia were administered to less than 5% of the population. We conducted a population-based study to evaluate the risk of thrombocytopenia and venous thromboembolism (VTE) with the BNT162b2, CoronaVac, and ChAdOx1 vaccines. Similarly, we assessed the risk of these outcome events following SARS-CoV-2 infection for comparison with the risk after vaccination.

## Materials and methods

### Study design

We used self-controlled case series (SCCS) method to measure the risks of thrombocytopenia and VTE after COVID-19 vaccination and SARS-CoV-2 infection. The SCCS determines the relative incidence of outcome for specific risk periods compared to control periods in individuals with the outcome of interest, in which persons act as their own controls and eliminate time-fixed confounding^21,22^. This method has been widely used and recommended for vaccine safety monitoring^23^.

### Data sources

Data on hospitalisations were retrieved from the Malaysian Health Data Warehouse, a national administration database that captures services and visits to healthcare facilities across Malaysia^24^. Hospital inpatient episodes contain details of patient information, admission and discharge dates, and diagnoses coded using the International Classification of Diseases 10th Revision (ICD-10). We used data from public hospitals, which cover approximately 70% of all hospital admissions in Malaysia^25^, due to poor data linkage ability for data from private hospitals. Individual-level COVID-19 vaccination data was extracted from the Malaysia Vaccine Administration System (MyVAS), a nationwide database that captured all COVID-19 vaccinations in Malaysia and provided information on the date, type, and dose of the administered vaccines. This database also includes details for individuals who received COVID-19 vaccination outside Malaysia, whose vaccination records were captured upon entry to Malaysia as part of the national requirements. Additionally, the national COVID-19 surveillance system which includes a registry of all patients with a confirmed SARS-CoV-2 infection in Malaysia provided information on COVID-19 diagnosis status (diagnostic test, date of positive test). These databases contain unique individual identification number which was used for data linkage using exact matching. Linked data were de-identified for further analysis.

### Exposure

The main exposure was COVID-19 vaccination, defined as receipt of BNT162b2, CoronaVac, and ChAdOx1. Other vaccines were less frequently used in the population in Malaysia and not included in the current analysis. The date of the vaccination was used as the exposure date. For individuals who received multiple vaccine doses during the observation period, each dose was considered separately. In Malaysia, the recommended vaccination dose schedule between the first and second dose is 21 days for BNT162b2 and CoronaVac and 4–12 weeks for ChAdOx1^26^. Individuals are eligible to receive a third dose, hereafter referred to as a booster dose, after a minimum of 90 days after completion of the primary dose schedule. Heterologous booster refers to receipt of a different vaccine brand than the primary dose series, while homologous booster refers to receipt of the same vaccine brand for the booster and primary dose series. Individuals were grouped by vaccine types and doses received. The secondary exposure was SARS-CoV-2 infection, defined as a positive test result for SARS-CoV-2. The date of tested positive was used as the exposure date.

### Outcomes

Study outcomes were hospital admission associated with thrombocytopenia and VTE, considered separately. VTE include a composite of pulmonary embolism, lower limb venous thrombosis, splanchnic thrombosis, and cerebral venous sinus thrombosis. Events were identified using ICD-10 codes from the diagnosis fields. The list of ICD-10 codes used for case identification is available in Appendix 1. Only the first event recorded during follow-up were included.

### Study population

We identified individuals admitted to hospitals with diagnoses of thrombocytopenia and VTE between 1 February 2021 and 28 February 2022, the study observation period, as cases. For each outcome, we excluded individuals who had a documented hospitalisation for the outcome diagnoses in the two years before the study period.

Vaccinated cohort comprised cases who had received at least one dose of BNT162b2, CoronaVac, or ChAdOx1. We excluded individuals from the vaccinated cohort if they (i) received any other COVID-19 vaccine brand, (ii) had a mixture of COVID-19 vaccine brands for the primary vaccination series (dose 1 and dose), and (iii) did not follow the dosing schedule. Those who had a positive SARS-CoV-2 test result in ≤ 30 days before or during admission for the outcome event were also excluded since COVID-19 can also increase the risk of outcome events^18,27^. We used 30 days to eliminate the risk during the acute infection period.

To study the association between SARS-CoV-2 infection and outcomes, SARS-CoV-2 infection cohort comprised of cases with a documented SARS-CoV-2 positive test result between 1 February 2021 and 28 February 2022. Individuals who received a COVID-19 vaccine dose in ≤ 30 days before or during admission for the outcome event were excluded from analysis of the infected cohort to eliminate risk potentially associated with the COVID-19 vaccination.

### Statistical analysis

**Figure 1:**
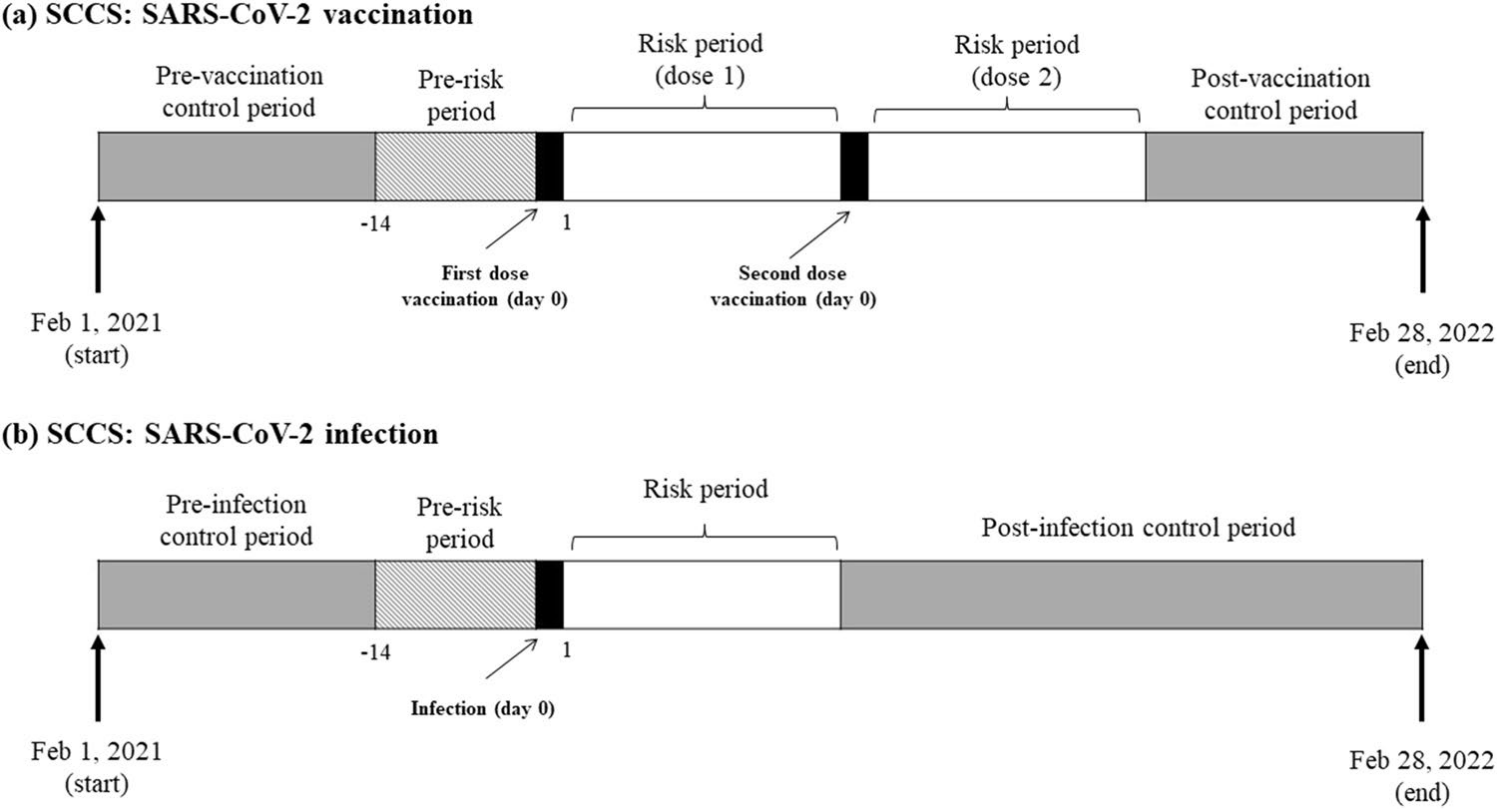
Follow-up of patients in self-controlled case series study for (**a**) COVID-19 vaccinated cohort and (**b**) COVID-19 infected cohort. Abbreviation: SCCS, self-controlled case series.

Analysis was conducted separately for each exposure and outcome of interest. Follow-up was from the start of the study period (1 February 2021) until 28 February 2022. The SCCS models were fitted using conditional Poisson regression model with an offset of the length of risk periods. Time windows were defined relative to the vaccination or infection date (Fig. 1). Exposure risk period was defined as 21 days following each exposure date (vaccination date or date of positive infection). The 21-day risk period was selected based on the vaccine dose schedule and published literature, which is considered sufficient to identify risk of acute events following vaccination or infection^18,28,29^. The day of exposure was considered separately (day 0). All other observation periods are considered as the reference period (control).

The models were adjusted for months in the observation period to account for potential factors associated with calendar time. Incidence rate ratios (IRRs) of events in the risk period relative to the control periods were calculated with the corresponding 95% confidence interval (CI) for each model. A 95% CI that did not include one indicates statistical significance. Each dose of vaccination was considered as a separate risk window to account for the dose effect. We excluded the booster dose from the SCCS model due to the low number of cases over the observation period to estimate them reliably. Follow-up for SCCS was to the end of the study period (28 February 2022) or censored upon receipt of a booster dose. The incidence of thrombocytopenia and VTE within 21 days after receiving a COVID-19 vaccine booster dose was presented descriptively as crude absolute risk over total doses administered.

Subgroup analyses were conducted by age and sex to explore any age or sex effect. Patient age was divided into the following categories: 18–39 years and ≥ 40 years. These age categories were used based on restricted recommendations for ChAdOx1 vaccine in those younger than 40 years old in several countries due to potential risk of blood clots and low platelets in the younger population^30,31^. VTE cases were also analysed by the types of thrombosis. A 14-day pre-risk period before exposure was removed from the control period and reported separately to account for potential bias if the outcome influenced the likelihood of exposure (event-dependent exposure). Another assumption of the SCCS method is that events must be independent of one another; therefore, analysis was restricted to only the first event. In a sensitivity analysis, we excluded fatal events to check for event-dependent censoring of observation time. Several other sensitivity analyses were conducted to check for robustness of the results: (i) including only pre-exposure period as the control period, and (ii) excluding individuals with any documented SARS-CoV-2 positive test before and during the study period. Cell numbers with values fewer than three were blinded and reported as less than three (< 3). Analyses were performed using STATA SE version 15 (StataCorp, College Station, Tx, USA).

### Ethical approval

This study was part of the project “Case-based monitoring of adverse events following COVID-19 vaccination (SAFECOVAC)” that received approval from the Medical Research and Ethics Committee, Ministry of Health Malaysia (NMRR-21-322-59745) which include waiver of informed consent due to the use of secondary data for this research.

## Results

**Figure 2:**
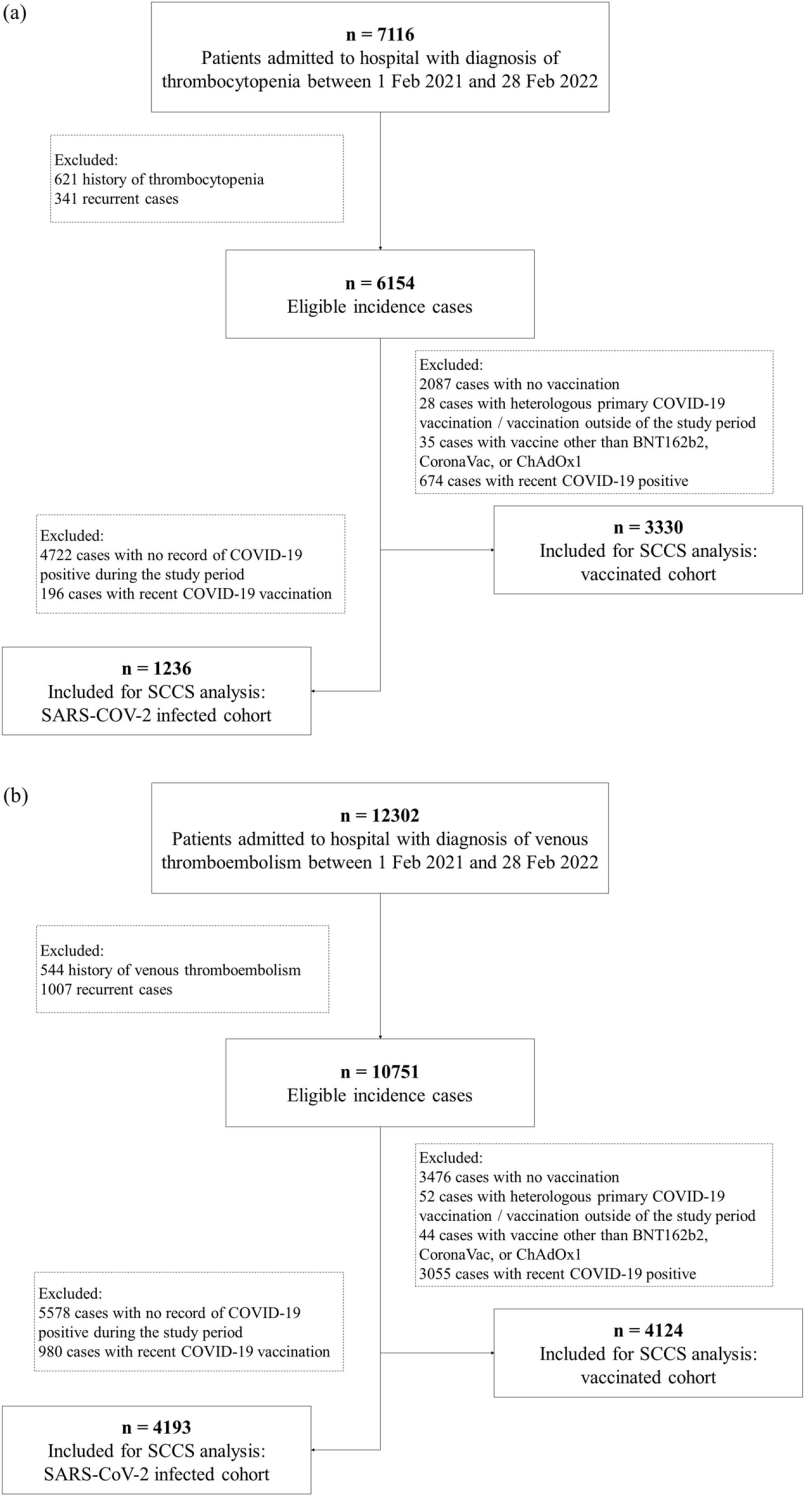
Flowchart of selection of (**a**) thrombocytopenia and (**b**) venous thromboembolism cases for analysis. Abbreviation: SCCS, self-controlled case series.

As of 28 February 2022, 90% of the adult population in Malaysia had been vaccinated against SARS-CoV-2 and over 14 million booster doses were administered to the population, while there were over 3 million (3,227,777) SARS-CoV-2 positive cases were recorded. Between 1 February 2021 and 28 February 2022, we identified a total of 7116 thrombocytopenia and 12,302 VTE that were hospitalised. The selection of cases for inclusion in the SCCS analysis is summarised in Fig. 2.

Table 1 shows characteristics of cases that met the eligibility criteria and grouped by COVID-19 vaccinated and infected cohorts. Among individuals who were vaccinated, there were 3330 thrombocytopenia and 4124 VTE cases. Majority (65%) had received BNT162b2 vaccine, while approximately 30% and 5% of them received CoronaVac and ChAdOx1 vaccines, respectively.

**Table 1:** Characteristics of cases admitted to hospitals between February 1, 2021 and February 28, 2022, stratified by SARS-CoV-2 vaccination and infection status.

	Vaccinated	SARS-CoV-2 infection
Thrombocytopenia		
N	3330	1236
Age in years, mean (SD)	51.5 (19.6)	50.6 (21.6)
Age in years, n (%)		
18–39	891 (26.8)	277 (22.4)
40–59	958 (28.8)	351 (28.4)
60+	1359 (40.8)	514 (41.6)
Male, n (%)	1754 (52.7)	685 (55.4)
Ethnicity, n (%)		
Malay	1901 (57.1)	724 (58.6)
Chinese	490 (14.7)	155 (12.5)
Indian	306 (9.2)	105 (8.5)
Others	541 (16.3)	209 (16.9)
Non-Malaysian, n (%)	91 (2.7)	40 (3.2)
Discharged alive, n (%)	3189 (95.0)	1094 (88.5)
COVID-19 vaccination		
BNT162b2	2202 (66.1)	–
CoronaVac	992 (29.8)	–
ChAdOx1	136 (4.1)	–
Venous thromboembolism		
N	4124	4193
Age in years, mean (SD)	52.3 (16.9)	52.6 (15.8)
Age in years, n (%)		
18–39	975 (23.6)	882 (21.0)
40–59	1520 (36.9)	1694 (40.4)
60+	1596 (38.7)	1589 (37.9)
Male, n (%)	1987 (48.2)	2179 (52.0)
Ethnicity, n (%)		
Malay	2461 (59.7)	2656 (63.3)
Chinese	646 (15.7)	526 (12.5)
Indian	379 (9.2)	363 (8.7)
Others	470 (11.4)	415 (9.9)
Non-Malaysian, n (%)	168 (4.1)	219 (5.2)
Discharged alive, n (%)	3783 (91.7)	3172 (75.6)
COVID-19 vaccination, n (%)		
BNT162b2	2670 (64.7)	–
CoronaVac	1279 (31.0)	–
ChAdOx1	175 (4.2)	–

Vaccinated refers to cases who received at least 1 dose of BNT162b2, CoronaVac, or ChAdOx1 vaccine.

*SD* standard deviation.

Of those included in the SARS-CoV-2 infected cohort, case counts were as follows: thrombocytopenia 1236, VTE 4193. Of the thrombocytopenia cases, 3189 cases (95.0%) from the vaccinated group were discharged alive compared to 88.5% in those infected with SARS-CoV-2. Among those who had VTE, cases that were discharged alive were 91.7% in the vaccinated group and 75.6% in those with SARS-CoV-2 infection.

### Thrombocytopenia

A total of 344 cases of thrombocytopenia occurred within 21 days after the first and second doses of BNT162, CoronaVac, and ChAdOx1 vaccines. The number of events and adjusted IRRs for each risk period by vaccine types and doses are summarised in Table 2. There was no increased risk of thrombocytopenia during 1 to 21 days after vaccination with BNT162b2 and CoronaVac compared to the control period. Results are consistent for both the first and second doses. The adjusted IRRs for ChAdOx1 were slightly elevated with values above 1, but the results were not significant (first dose, IRR 1.36; 95% CI 0.67–2.78; second dose, IRR 1.45; 95% CI 0.68–3.10). None of the strata showed a significantly higher risk of thrombocytopenia after vaccination when the risk period was subdivided into separate weeks.

**Table 2:** Association between SARS-CoV-2 vaccination or infection and outcome events using self-controlled case series analysis, adjusted for calendar month between 1 February 2021 and 28 February 2022.

	Thrombocytopenia			Venous thromboembolism		
	Event	Patient-years	IRR (95% CI)	Event	Patient-years	IRR (95% CI)
BNT162b2	(N = 2059)			(N = 2500)		
Control period	1794	1918.35	1.00	2177	2321.86	1.00
1st dose						
Day 1–21	103	121.21	0.83 (0.68 to 1.02)	153	147.43	0.93 (0.78 to 1.10)
Subinterval						
Day 1–7	36	42.13	0.83 (0.60 to 1.16)	38	51.11	0.67 (0.48 to 0.92)
Day 8–14	41	42.03	0.95 (0.70 to 1.30)	59	51.01	1.04 (0.80 to 1.35)
Day 15–21	26	42.03	0.69 (0.47 to 1.02)	56	45.31	1.11 (0.85 to 1.45)
2nd dose						
Day 1–21	117	120.39	0.96 (0.79 to 1.16)	126	141.50	0.80 (0.66 to 0.96)
Subinterval						
Day 1–7	35	40.15	0.86 (0.61 to 1.21)	41	47.22	0.78 (0.57 to 1.06)
Day 8–14	40	40.14	0.98 (0.71 to 1.35)	38	47.18	0.72 (0.52 to 1.00)
Day 15–21	42	40.10	1.03 (0.75 to 1.40)	47	47.10	0.90 (0.67 to 1.20)
CoronaVac	(N = 985)			(N = 1274)		
Control period	867	829.86		1072	1076.63	1.00
1st dose						
Day 1–21	48	54.73	0.78 (0.57 to 1.08)	100	70.61	0.99 (0.79 to 1.25)
Subinterval						
Day 1–7	13	19.01	0.61 (0.35 to 1.08)	26	24.51	0.77 (0.51 to 1.15)
Day 8–14	20	19.01	0.94 (0.59 to 1.49)	33	24.49	0.94 (0.65 to 1.35)
Day 15–21	15	16.70	0.79 (0.47 to 1.35)	41	21.62	1.28 (0.92 to 1.77)
2nd dose						
Day 1–21	57	54.16	0.92 (0.68 to 1.23)	85	141.50	0.79 (0.62 to 1.00)
Subinterval						
Day 1–7	11	18.05	0.53 (0.29 to 0.98)	23	22.83	0.65 (0.43 to 1.00)
Day 8–14	25	18.05	1.21 (0.80 to 1.84)	28	22.83	0.77 (0.53 to 1.14)
Day 15–21	21	18.05	1.01 (0.65 to 1.59)	34	22.81	0.93 (0.65 to 1.32)
ChAdOx1	(N = 130)			(N = 172)		
Control period	111	119.43	1.00	145	153.44	1.00
1st dose						
Day 1–21	10	7.81	1.36 (0.67 to 2.78)	17	10.05	1.54 (0.88 to 2.73)
Subinterval						
Day 1–7	3	2.61	1.30 (0.40 to 4.28)	< 3	3.35	**
Day 8–14	< 3	2.61	**	10	3.35	2.74 (1.37 to 5.49)
Day 15–21	6	2.60	2.27 (0.95 to 5.45)	5	3.35	1.30 (0.51 to 3.27)
2nd dose						
Day 1–21	9	7.05	1.45 (0.68 to 3.10)	9	8.62	0.61 (0.30 to 1.22)
Subinterval						
Day 1–7	< 3	2.36	**	< 3	2.87	**
Day 8–14	3	2.36	1.50 (0.45 to 4.96)	3	2.87	0.60 (0.19 to 1.92)
Day 15–21	4	2.34	1.75 (0.61 to 5.00)	4	2.87	0.82 (0.30 to 2.25)
SARS-CoV-2 infection	(N = 1236)			(N = 4193)		
Control period	496	1211.13	1.00	964	4101.50	1.00
Day 1–21	381	68.42	15.52 (13.38 to 18.00)	1929	238.77	29.84 (27.45 to 32.44)
Subinterval						
Day 1–7	317	23.34	38.09 (32.64 to 44.46)	1436	80.11	64.99 (59.60 to 70.89)
Day 8–14	32	22.80	3.87 (2.69 to 5.57)	307	79.57	13.99 (12.26 to 15.97)
Day 15–21	32	22.28	3.91 (2.72 to 5.63)	186	79.09	8.57 (7.30 to 10.06)

Control period include days from 1 February 2021 to 28 February 2022 outside of the risk period (1–21 days post exposure), pre-risk period (− 14 to − 1 day before exposure), and day of exposure (day 0).

*CI* confidence interval, *IRR* incidence rate ratio.

**Value not computed for event count less than 3.

Thrombocytopenia was observed in 381 patients within 21 days following a positive SARS-CoV-2 test, resulting in an adjusted IRR of 15.52 (95% CI 13.38–18.00) (Table 2). The risk was highest during the first week following infection (IRR 38.09; 95% CI 32.64–44.46).

### Venous thromboembolism

The risk of VTE was found to be not significantly increased during the 1–21 days after both the first and second doses of BNT162b2, CoronaVac, and ChAdOx1 (Table 2). There was some evidence of increased risk of VTE associated with ChAdOx1 when the risk period was subdivided by weeks since vaccination, where the risk estimates during day 8–14 after the first dose of ChAdOx1 was 2.74 (1.37–5.49).

In the 21-day risk period following SARS-CoV-2 infection, there were 1929 cases of VTE. A significantly increased risk of VTE was observed during the risk period (day 1–21) compared to the control period with IRR of 29.84 (95% CI 27.45–32.44). Analysis by week showed that the risk was highest during the first week following infection (IRR 64.99; 95% CI 59.60–70.89).

### Subgroup and sensitivity analyses

Consistent with the results of the main analysis, subgroup analyses stratified by age groups and sex did not show significant association between the outcome events and vaccination with BNT162b2, CoronaVac, or ChAdOx1 within the 21-day risk period after vaccination (Table 3). The risk of VTE after the first dose of ChAdOx1 was two-fold higher in females than males, but it was not statistically significant. Increased risk of thrombocytopenia and VTE following SARS-CoV-2 infection was similarly observed when analysis was stratified by age and sex but we did not observe any difference in the risk across the subgroup. Subgroup analysis by types of VTE did not result in significant IRR (Appendix 2).

**Table 3:** Subgroup analysis by age and sex for association between SARS-CoV-2 vaccination or infection and outcome events using self-controlled case series analysis, adjusted for calendar month between 1 February 2021 and 28 February 2022.

	BNT162b2		CoronaVac		ChAdOx1		SARS-CoV-2	
	Event	IRR (95% CI)	Event	IRR (95% CI)	Event	IRR (95% CI)	Event	IRR (95% CI)
Thrombocytopenia								
18–39 years								
Control period	529	1.00	201	1.00	36	1.00	138	1.00
1st dose/infection, day 1–21	23	0.57 (0.37 to 0.89)	13	0.82 (0.43 to 1.56)	3	2.68 (0.65 to 11.07)	85	12.37 (9.18 to 16.66)
2nd dose, day 1–21	33	0.84 (0.57 to 1.21)	8	0.55 (0.25 to 1.19)	< 3	**	–	–
≥ 40 years								
Control period	1156	1.00	666	1.00	75	1.00	297	1.00
1st dose/infection, day 1–21	73	0.91 (0.71 to 1.17)	35	0.76 (0.52 to 1.11)	7	1.47 (0.62 to 3.51)	282	19.19 (15.99 to 23.04)
2nd dose, day 1–21	79	1.00 (0.79 to 1.27)	49	1.05 (0.76 to 1.46)	8	1.69 (0.74 to 3.83)	–	–
Male								
Control period	916	1.00	460	1.00	63	1.00	254	1.00
1st dose/infection, day 1–21	51	0.82 (0.61 to 1.10)	27	0.85 (0.56 to 1.31)	6	1.29 (0.51 to 3.22)	222	16.79 (13.78 to 20.45)
2nd dose, day 1–21	66	1.06 (0.82 to 1.38)	40	1.20 (0.84 to 1.72)	5	1.36 (0.50 to 3.73)	–	
Female								
Control period	878	1.00	407	1.00	48	1.00	242	1.00
1st dose/infection, day 1–21	52	0.84 (0.63 to 1.12)	21	0.70 (0.43 to 1.13)	4	1.51 (0.48 to 4.71)	159	14.19 (11.32 to 17.78)
2nd dose, day 1–21	51	0.84 (0.63 to 1.13)	17	0.58 (0.35 to 0.99)	4	1.56 (0.49 to 5.02)	–	–
Venous thromboembolism								
18–39 years								
Control period	563	1.00	227	1.00	27	1.00	227	1.00
1st dose/infection, day 1–21	39	0.87 (0.62 to 1.23)	18	0.76 (0.45 to 1.30)	3	1.01 (0.24 to 4.16)	445	29.05 (24.12 to 35.00)
2nd dose, day 1–21	28	0.67 (0.45 to 0.99)	18	0.70 (0.41 to 1.19)	2	0.51 (0.11 to 2.39)	–	–
≥ 40 years								
Control period	1585	1.00	845	1.00	118	1.00	723	1.00
1st dose/infection, day 1–21	113	0.92 (0.76 to 1.12)	82	1.04 (0.80 to 1.34)	14	1.62 (0.86 to 3.04)	1477	30.31 (27.57 to 33.32)
2nd dose, day 1–21	96	0.82 (0.67 to 1.02)	67	0.79 (0.61 to 1.03)	7	0.61 (0.28 to 1.35)	–	–
Male								
Control period	993	1.00	528	1.00	88	1.00	466	1.00
1st dose/infection, day 1–21	77	1.01 (0.80 to 1.29)	56	1.05 (0.78 to 1.43)	10	1.23 (0.60 to 2.52)	1032	32.42 (28.84 to 36.45)
2nd dose, day 1–21	67	0.94 (0.73 to 1.21)	46	0.85 (0.62 to 1.18)	6	0.60 (0.25 to 1.42)	–	–
Female								
Control period	1184	1.00	544	1.00	56	1.00	498	1.00
1st dose/infection, day 1–21	76	0.86 (0.68 to 1.09)	44	0.94 (0.67 to 1.33)	7	2.53 (0.97 to 6.60)	897	27.40 (24.30 to 30.90)
2nd dose, day 1–21	59	0.69 (0.52 to 0.90)	39	0.72 (0.51 to 1.03)	3	0.60 (0.18 to 2.03)	–	–

Control period include days from 1 February 2021 to 28 February 2022 outside of the risk period (1–21 days post exposure), pre-risk period (− 14 to − 1 day before exposure), and day of exposure (day 0).

*CI* confidence interval, *IRR* incidence rate ratio.

**Value not computed for event count less than 3.

Results of the sensitivity analyses remain largely similar to the main analysis and confirm the robustness of the results. Exclusion of fatal events from the SCCS model showed no association between COVID-19 vaccination and the risk of thrombocytopenia and VTE (Appendix 3). When we restricted the control period to only include the pre-exposure period (Appendix 4), the risks increased slightly particularly for thrombocytopenia following the second dose of BNT162b2 and CoronaVac vaccines, but stayed non-significant. Exclusion of cases with any history of COVID-19 diagnosis (Appendix 5) showed estimates that were consistent with the main analysis, but with wider confidence intervals due to the smaller number of cases included.

### Risk of events after COVID-19 vaccine booster dose

Table 4 shows the number of thrombocytopenia and VTE that were observed following the administration of COVID-19 vaccine booster dose. Overall, there were 58 cases of thrombocytopenia and 67 cases of VTE within the first 21 days after receipt of the booster dose. The absolute risk of the event ranged from three to six cases for every one million booster vaccine doses administered. Most cases were reported among recipients of BNT162b2 as booster dose irrespective of the primary vaccination series. The absolute risks of thrombocytopenia within 21 days after booster vaccination for heterologous and homologous booster groups were 4.5 (95% CI 3.1–6.3) and 3.4 (95% CI 2.2–5.0) per 1 million doses, respectively. For VTE, the absolute risk in heterologous booster was 4.9 (95% CI 3.5–6.8) compared to 4.2 (95% CI 2.8–6.0) in homologous group.

**Table 4:** Thrombocytopenia and venous thromboembolism following COVID-19 vaccine booster dose.

Primary series/booster vaccine	No. of booster doses	Event (1–21 day)	Crude risk per 100,000 doses (95% CI)	Event (> 21 day)	Crude risk per 100,000 doses (95% CI)
Thrombocytopenia					
BNT162b2					
BNT162b2	5,312,389	17	0.32 (0.19 to 0.51)	44	0.83 (0.60 to 1.11)
CoronaVac	16,003	–	–	–	–
ChAdOx1	334,895	< 3	**	< 3	**
CoronaVac					
BNT162b2	5,966,652	31	0.52 (0.35 to 0.74)	49	0.82 (0.61 to 1.09)
CoronaVac	1,103,513	4	0.36 (0.10 to 0.93)	3	0.27 (0.06 to 0.79)
ChAdOx1	465,243	–	–	< 3	**
ChAdOx1					
BNT162b2	713,579	< 3	**	–	–
CoronaVac	2461	–	–	–	–
ChAdOx1	730,109	3	0.41 (0.08 to 1.20)	–	–
Venous thromboembolism					
BNT162b2					
BNT162b2	5,312,389	30	0.56 (0.38 to 0.81)	41	0.77 (0.55 to 1.05)
CoronaVac	16,003	–	–	–	–
ChAdOx1	334,895	< 3	**	< 3	**
CoronaVac					
BNT162b2	5,966,652	33	0.55 (0.38 to 0.78)	61	1.02 (0.78 to 1.31)
CoronaVac	1,103,513	–	–	5	0.45 (0.15 to 1.06)
ChAdOx1	465,243	–	–	< 3	**
ChAdOx1					
BNT162b2	713,579	3	0.42 (0.09 to 1.23)	< 3	**
CoronaVac	2461	–	–	–	–
ChAdOx1	730,109	–	–	–	–

*CI* confidence interval, *NA* not available.

**Value not computed for event count less than 3.

## Discussion

This study estimated the risk of hospitalisation for thrombocytopenia and VTE following SARS-CoV-2 vaccination and infection. Risk estimates for both thrombocytopenia and VTE during the 21-day risk period were > 1 following exposure to ChAdOx1 vaccine, but were not statistically significant. No increased risk was observed following BNT162b2 and CoronaVac. A substantially higher risk of both thrombocytopenia and VTE was observed after SARS-CoV-2 infection.

Previous studies showed an increased risk of thrombocytopenia, VTE, and cerebral venous sinus thrombosis following COVID-19 vaccination, particularly with ChAdOx1^12,14,32,33^. Recently, Berild et al.^13^ reported elevated risk of thrombocytopenia and thromboembolic events after ChAdOx1 in an SCCS analysis that pooled data from Norway, Finland, and Denmark. Notably, these studies only studied the first dose of vaccine due to the limited number of people receiving second dose during the study period. Our findings included analysis by first and second dose which showed that there was an indication of slightly increased risk of thrombocytopenia and VTE among people receiving ChAdOx1 vaccine, but the association was not statistically significant. We did find a significant association between ChAdOx1 and VTE during day 8–14 after the first dose (IRR 2.74; 95% CI 1.37–5.49), which corresponds to other studies that found the risk to be higher around week 2 after vaccination^12,34^. Nevertheless, the numbers were small and further confirmation is needed. Our study cohort represents an Asian population of multi-ethnicity which was slightly different than previous studies that were predominantly Caucasian. Furthermore, the vaccination policy in Malaysia has to be taken into account whereby ChAdOx1 vaccine was initially given as voluntary opt-in due to safety concerns during the roll-out^3^. ChAdOx1 vaccine was well-received among the younger population due to its availability before their originally scheduled vaccination appointments^3^. Therefore, people who received ChAdOx1 represent a younger and likely healthier population. In this study, we did not find significant association between BNT162b2 and the outcome events. This broadly echoes other studies that reported no statistically significant risk for thrombocytopenia and thromboembolic events in the 3–4 weeks following BNT162b2 vaccination^14,33^. Our findings further showed no evidence of association for such events among people who received CoronaVac which aligned with published studies from Hong Kong^35,36^.

Many countries, including Malaysia, introduced a booster dose of COVID-19 vaccine in fully vaccinated recipients to enhance waning induced immunity and immunity against new SARS-CoV-2 variants^37^. In our study, thrombocytopenia and VTE that occurred during 1 to 21 days after receipt of COVID-19 vaccine booster accounted for less than ten cases for every one million booster doses administered. Cases were mostly reported for BNT162b2 which corresponds to the country’s COVID-19 vaccine booster coverage since BNT162b2 was the primary vaccine administered as a booster shot to the population while another vaccine type was offered as an alternative to BNT162b2. Our findings showed comparable safety profiles between those who received homologous and heterologous booster combinations, which provides reassurance on the safety of these booster combinations.

While the concern of post-vaccination thrombosis is one of the reasons for vaccine hesitancy in many, the present analysis has shown contrarily that the risk of developing these thrombotic events following SARS-CoV2 infection itself was way higher than the risk from COVID-19 vaccination. Our findings hence support other studies where high rates of thrombocytopenia and VTE were encountered in patients with SARS-CoV2 infection, than after a COVID-19 vaccine in the same population^12,38–40^. The reason for this is that COVID-19 disease may itself be associated with coagulation dysfunction which predisposes patients to an elevated risk of thromboembolism^40,41^. However, the specific mechanism(s) for the increased incidence of venous thrombosis among patients with SARS-CoV2 infection has not been fully elucidated. Importantly, the risks of both thrombocytopenia and VTE after COVID-19 vaccination were much lower than those associated with SARS-CoV-2 infection.

### Strength and limitation

Our study provided real-world evidence on the safety of COVID-19 vaccination with direct comparisons between the three different vaccine platforms within the same population. Particularly, we provided evidence on the safety profiles of inactivated CoronaVac vaccine, which are still limited compared to other platforms despite accounting for nearly 50% of the COVID-19 vaccine doses delivered globally^42^. Compared to existing evidence from studies conducted mainly in Western countries, our cohort consists of an Asian population administered different types of COVID-19 vaccines, including administration of both homologous and heterologous booster doses. We used large, nationwide database to provide population-based safety data on the risks associated with both COVID-19 vaccination and infection. Linkage to national databases of COVID-19 vaccination and COVID-19 cases minimises the bias from misclassifying exposure.

There are several limitations in this study. First, the outcome events were based on hospital admissions. We did not include cases that were treated in primary care, outpatient, or those who died before being admitted. Second, we did not include data from private hospitals. Nevertheless, public hospitals cover approximately 70% of all hospital admissions in the country^43^; therefore, we expect the cases that were not captured to be relatively small. Third, case ascertainment was based on diagnosis codes of hospital records which might overestimate the risk without including other laboratory parameters to define and validate the case. Fourth, we do not have data on medication history or pharmacological treatment provided during hospitalisation. Therefore, we cannot adjust for inherent factors pertaining to individuals who might be on certain treatments that could affect the risk profile. Similarly, we do not have data on individuals’ comorbidities and therefore, we are unable to account for the effect of existing medical conditions on the association measured in this study. To minimise this bias, we excluded individuals who had experienced the outcome in the 2 years before the index hospitalisation and included only the first event that occurred during the study period for analysis. Next, with the use of secondary databases, we cannot completely rule out misclassification bias and unmeasured confounding, such as smoking, alcohol consumption, body mass index, or disease severity. These factors are known to be common risk factors for the outcome, but we did not have the information in the database that we used. Therefore, further studies will have to address these information gaps and risk factors not adjusted for in this study. Lastly, some of the subgroup analyses present a small sample size and results should be interpreted with caution.

### Policy implications

The finding on the risk of thrombocytopenia and thrombotic complications after COVID-19 vaccines and after SARS-CoV-2 infection will be useful in the risk–benefit assessment for vaccine-related policies or purposes and public health decision-making as well as in providing quantification of the risk of thrombotic events associated with COVID-19 vaccines to the general public. This also emphasises the need for clinicians to implement thromboprophylaxis protocols to reduce the risk of thromboembolism among SARS-CoV-2 infected individuals. The findings of the present study also provided some insights into the safety profile of booster doses of COVID-19 vaccination. This information may help address vaccine hesitancy, which is a key challenge for public health regarding containing infectious diseases and pandemic prevention.

## Conclusion

Among our study population, the risks of thrombocytopenia and VTE were not increased during 1–21 days after COVID-19 vaccination compared with the control period. The risks of these events were higher after vaccination with ChAdOx1 compared to BNT162b2 and CoronaVac, although the increase in risk was not significant. The rates of both thrombocytopenia and VTE after booster doses were generally low after either vaccine. However, SARS-CoV-2 infections were associated with a significantly increased risk of both thrombocytopenia and VTE in the same population. The benefit-risk profiles of COVID-19 vaccination pertaining to these events should be interpreted in light of current findings to guide decision-making for vaccination to protect against complications of SARS-CoV-2 infection.

### Supplementary Information

Supplementary Information.

## Data Availability

The data that supports the findings of this study are available within the article and its supplementary materials. Access to datasets is provided by the corresponding data custodians for analysis for this study, but we have no permission to make generated datasets available. Malaysia COVID-19 vaccine administration data are available at https://github.com/MoH-Malaysia/covid19-public, redacted of personal identifying information.
