# Supplementary material for: Thrombocytopenia and venous thromboembolic events after BNT162b2, CoronaVac, ChAdOx1 vaccines and SARS-CoV-2 infection: a self-controlled case series study
Source: Sci Rep. 2023 Nov 22;13:20471. doi: 10.1038/s41598-023-47486-x (PMC10665324; doi:10.1038/s41598-023-47486-x)
Supplement: Supplementary file 1 — Supplementary Information. [file 41598_2023_47486_MOESM1_ESM.docx]

**SUPPLEMENTARY MATERIAL**

**TITLE:** Thrombocytopenia and venous thromboembolic events after BNT162b2, CoronaVac, ChAdOx1 vaccines and SARS-CoV-2 infection: a self-controlled case series study

**AUTHOR LIST:**

Norazida Ab Rahman ^a,^ *, Ming Tsuey Lim ^a^, Fei Yee Lee ^b^, Wee Kee Wo ^c^, Hee Sheong Yeoh ^c^, Kalaiarasu M Peariasamy ^a^, Sheamini Sivasampu ^a^, for the SAFECOVAC study group

**^a^** Institute for Clinical Research, National Institutes of Health, Selangor, Malaysia

**^b^** Clinical Research Centre, Selayang Hospital, Ministry of Health, Selangor, Malaysia

**^c^** National Pharmaceutical Regulatory Agency, Ministry of Health, Selangor, Malaysia

**CORRESPONDENCE:**

Norazida Ab Rahman

Institute for Clinical Research

Block B4, National Institutes of Health

No 1, Jalan Setia Murni U13/52

40170 Shah Alam, Selangor, Malaysia

Tel: +603 33628824

Email: [norazida@crc.gov.my](mailto:norazida@crc.gov.my)

**Appendix 1 List of diagnoses codes by International Classification of Diseases 10^th^ Revision (ICD-10)**

**Appendix 2 Subgroup analysis by types of venous thromboembolism in the self-controlled case-series**

**Appendix 3 Sensitivity analysis for self-controlled case series with exclusion of fatal events**

**Appendix 4 Sensitivity analysis for self-controlled case series with pre-exposure as control period**

**Appendix 5 Sensitivity analysis for self-controlled case series with exclusion of cases with any history of SARS-CoV-2 infection before and during observation period**

**Appendix 6 SAFECOVAC study group members, non-author collaborators**

Appendix 1 List of diagnoses codes by International Classification of Diseases 10^th^ Revision (ICD-10)

| **Outcome** | **ICD-10 code** |
| --- | --- |
| Thrombocytopenia | M31.1, D69.3, D69.4, D69.5, D69.6 |
| Venous thromboembolism | I80, I80.1, I80.2, I80.29, I80.3, I82.2, I82.8, I82.9, I26, I26.9, I81, I82.0, I82.1, I82.3, I67.6 |
| *Pulmonary embolism* | I26, I26.9 |
| *Lower limb venous thrombosis* | I80, I80.1, I80.2, I80.29, I80.3 |
| *Splanchnic thrombosis* | I81, I82.0, I82.1, I82.3 |
| *Other venous thrombosis* | I82.2, I82.8, I82.89, I82.9 |
| *Cerebral venous thrombosis* | I67.6 |

Appendix 2 Subgroup analysis by types of venous thromboembolism in the self-controlled case-series

|  |  | **BNT162b2** |  | **CoronaVac** |  | **ChAdOx1** |  | **SARS-CoV-2** |
| --- | --- | --- | --- | --- | --- | --- | --- | --- |
|  | **Event** | **IRR (95% CI)** | **Event** | **IRR (95% CI)** | **Event** | **IRR (95% CI)** | **Event** | **IRR (95% CI)** |
| **Pulmonary embolism** |  |  |  |  |  |  |  |  |
| Control period | 1141 | 1.00 | 644 | 1.00 | 79 | 1.00 | 615 | 1.00 |
| ***1^st^ dose / infection*** |  |  |  |  |  |  |  |  |
| Day 1-21 | 89 | 0.88 (0.70 to 1.10) | 75 | 1.02 (0.78 to 1.33) | 12 | 1.62 (0.81 to 3.24) | 1852 | 41.53 (37.74 to 45.70) |
| ***2^nd^ dose*** |  |  |  |  |  |  |  |  |
| Day 1-21 | 64 | 0.68 (0.53 to 0.88) | 55 | 0.69 (0.52 to 0.93) | 6 | 0.61 (0.26 to 1.43) | - | - |
|  |  |  |  |  |  |  |  |  |
| **Lower limb venous thrombosis** |  |  |  |  |  |  |  |  |
| Control period | 496 | 1.00 | 236 | 1.00 | 33 |  | 187 | 1.00 |
| ***1^st^ dose / infection*** |  |  |  |  |  |  |  |  |
| Day 1-21 | 36 | 1.11 (0.78 to 1.58) | 14 | 1.01 (0.55 to 1.84) | <3 | ** | 51 | 4.70 (3.37 to 6.55) |
| ***2^nd^ dose*** |  |  |  |  |  |  |  |  |
| Day 1-21 | 27 | 0.85 (0.57 to 1.27) | 19 | 1.32 (0.78 to 2.21) | <3 | ** | - | - |
|  |  |  |  |  |  |  |  |  |
| **Other venous thrombosis** |  |  |  |  |  |  |  |  |
| Control period | 481 | 1.00 | 172 | 1.00 | 29 | 1.00 | 167 | 1.00 |
| ***1^st^ dose / infection*** |  |  |  |  |  |  |  |  |
| Day 1-21 | 25 | 0.81 (0.53 to 1.22) | 13 | 0.91 (0.48 to 1.72) | <3 | ** | 30 | 3.97 (2.62 to 6.02) |
| ***2^nd^ dose*** |  |  |  |  |  |  |  |  |
| Day 1-21 | 29 | 1.00 (0.68 to 1.47) | 10 | 0.81 (0.41 to 1.62) | <3 | ** | - | - |
|  |  |  |  |  |  |  |  |  |

Control period include days from 1 February 2021 to 28 February 2022 outside of the risk period (1-21 days post exposure), pre-risk period (-14 to -1 day before exposure), and day of exposure (day 0). Analysis adjusted for calendar months. There were less than 10 cases of cerebral venous sinus thrombosis that occurred during the risk period for both post-vaccination and post-infection period. Abbreviations: CI, confidence interval; IRR, incidence rate ratio. ** Value not computed for event count less than 3.

Appendix 3 Sensitivity analysis for self-controlled case series with exclusion of fatal events

|  |  | **BNT162b2** |  | **CoronaVac** |  | **ChAdOx1** |  | **SARS-CoV-2** |
| --- | --- | --- | --- | --- | --- | --- | --- | --- |
|  | **Event** | **IRR (95% CI)** | **Event** | **IRR (95% CI)** | **Event** | **IRR (95% CI)** | **Event** | **IRR (95% CI)** |
| **Thrombocytopenia** |  |  |  |  |  |  |  |  |
| Control period | 1708 | 1.00 | 822 | 1.00 | 108 | 1.00 | 481 | 1.00 |
| ***1^st^ dose / infection*** |  |  |  |  |  |  |  |  |
| Day 1-21 | 98 | 0.83 (0.67 to 1.03) | 44 | 0.75 (0.53 to 1.04) | 10 | 1.39 (0.68 to 2.84) | 327 | 14.11 (12.07 to 16.49) |
| Day 1-7 | 34 | 0.82 (0.58 to 1.16) | 13 | 0.63 (0.36 to 1.12) | 3 | 1.32 (0.40 to 4.36) | 270 | 34.27 (29.11 to 40.36) |
| Day 8-14 | 38 | 0.93 (0.67 to 1.29) | 18 | 0.87 (0.54 to 1.42) | <3 | ** | 27 | 3.46 (2.33 to 5.13) |
| Day 15-21 | 26 | 0.73 (0.49 to 1.08) | 13 | 0.72 (0.41 to 1.27) | 6 | 2.32 (0.96 to 5.57) | 30 | 3.89 (2.67 to 5.67) |
| ***2^nd^ dose*** |  |  |  |  |  |  |  |  |
| Day 1-21 | 106 | 0.91 (0.75 to 1.12) | 53 | 0.91 (0.67 to 1.24) | 8 | 1.32 (0.59 to 2.92) | - | - |
| Day 1-7 | 33 | 0.85 (0.60 to 1.21) | 10 | 0.51 (0.27 to 0.97) | <3 | ** | - | - |
| Day 8-14 | 38 | 0.98 (0.71 to 1.36) | 22 | 1.14 (0.73 to 1.77) | <3 | ** | - | - |
| Day 15-21 | 35 | 0.91 (0.65 to 1.27) | 21 | 1.09 (0.69 to 1.71) | 4 | 1.81 (0.63 to 5.18) | - | - |
|  |  |  |  |  |  |  |  |  |
| **Venous thromboembolism** |  |  |  |  |  |  |  |  |
| Control period | 2048 | 1.00 | 962 | 1.00 | 133 | 1.00 | 881 | 1.00 |
| ***1^st^ dose / infection*** |  |  |  |  |  |  |  |  |
| Day 1-21 | 130 | 0.85 (0.70 to 1.01) | 89 | 0.97 (0.76 to 1.23) | 15 | 1.49 (0.82 to 2.72) | 1449 | 25.31 (23.11 to 27.72) |
| Day 1-7 | 28 | 0.52 (0.36 to 0.76) | 24 | 0.77 (0.51 to 1.17) | <3 | ** | 1040 | 53.25 (48.40 to 58.60) |
| Day 8-14 | 55 | 1.03 (0.79 to 1.36) | 29 | 0.90 (0.62 to 1.33) | 8 | 2.38 (1.11 to 5.14) | 250 | 12.91 (11.17 to 14.92) |
| Day 15-21 | 47 | 1.00 (0.74 to 1.34) | 36 | 1.24 (0.88 to 1.76) | 5 | 1.42 (0.56 to 3.62) | 159 | 8.31 (6.99 to 9.88) |
| ***2^nd^ dose*** |  |  |  |  |  |  |  |  |
| Day 1-21 | 112 | 0.76 (0.63 to 0.92) | 74 | 0.77 (0.60 to 0.99) | 9 | 0.65 (0.32 to 1.32) | - | - |
| Day 1-7 | 35 | 0.71 (0.51 to 0.99) | 18 | 0.57 (0.35 to 0.92) | <3 | NA | - | - |
| Day 8-14 | 33 | 0.67 (0.47 to 0.95) | 24 | 0.74 (0.49 to 1.13) | 3 | 0.64 (0.20 to 2.06) | - | - |
| Day 15-21 | 44 | 0.90 (0.67 to 1.22) | 32 | 0.99 (0.69 to 1.42) | 4 | 0.89 (0.32 to 2.45) | - | - |

Control period include days from 1 February 2021 to 28 February 2022 outside of the risk period (1-21 days post exposure), pre-risk period (-14 to -1 day before exposure), and day of exposure (day 0). Analysis adjusted for calendar months. Abbreviations: CI, confidence interval; IRR, incidence rate ratio. ** Value not computed for event count less than 3.

Appendix 4 Sensitivity analysis for self-controlled case series with pre-exposure as control period

|  |  | **BNT162b2** |  | **CoronaVac** |  | **ChAdOx1** |  | **SARS-CoV-2** |
| --- | --- | --- | --- | --- | --- | --- | --- | --- |
|  | **Event** | **IRR (95% CI)** | **Event** | **IRR (95% CI)** | **Event** | **IRR (95% CI)** | **Event** | **IRR (95% CI)** |
| **Thrombocytopenia** |  |  |  |  |  |  |  |  |
| Control period | 964 | 1.00 | 410 | 1.00 | 68 | 1.00 | 331 | 1.00 |
| ***1^st^ dose / infection*** |  |  |  |  |  |  |  |  |
| Day 1-21 | 103 | 0.94 (0.74 to 1.20) | 48 | 0.87 (0.59 to 1.29) | 10 | 1.39 (0.66 to 2.93) | 381 | 12.58 (9.88 to 16.02) |
| Day 1-7 | 36 | 0.93 (0.65 to 1.33) | 13 | 0.69 (0.38 to 1.25) | 3 | 1.33 (0.40 to 4.50) | 317 | 28.27 (22.28 to 35.87) |
| Day 8-14 | 41 | 1.08 (0.77 to 1.52) | 20 | 1.06 (0.64 to 1.78) | <3 | ** | 32 | 2.83 (1.86 to 4.26) |
| Day 15-21 | 26 | 0.79 (0.52 to 1.19) | 15 | 0.92 (0.51 to 1.64) | 6 | 2.34 (0.94 to 5.81) | 32 | 2.81 (1.86 to 4.25) |
| ***2^nd^ dose*** |  |  |  |  |  |  |  |  |
| Day 1-21 | 117 | 1.16 (0.90 to 1.50) | 57 | 1.08 (0.71 to 1.66) | 9 | 1.90 (0.71 to 5.14) | - | - |
| Day 1-7 | 35 | 1.02 (0.70 to 1.48) | 11 | 0.64 (0.33 to 1.26) | <3 | ** | - | - |
| Day 8-14 | 40 | 1.19 (0.83 to 1.71) | 25 | 1.48 (0.87 to 2.49) | 3 | ** | - | - |
| Day 15-21 | 42 | 1.29 (0.90 to 1.85) | 21 | 1.25 (0.71 to 2.19) | 4 | 2.51 (0.72 to 8.77) | - | - |
|  |  |  |  |  |  |  |  |  |
| **Venous thromboembolism** |  |  |  |  |  |  |  |  |
| Control period | 1170 | 1.00 | 467 | 1.00 | 133 | 1.00 | 437 | 1.00 |
| ***1^st^ dose / infection*** |  |  |  |  |  |  |  |  |
| Day 1-21 | 153 | 0.97 (0.80 to 1.19) | 100 | 0.99 (0.75 to 1.30) | 17 | 1.88 (1.02 to 3.47) | 1929 | 46.23 (38.21 to 55.93) |
| Day 1-7 | 38 | 0.70 (0.50 to 0.98) | 26 | 0.78 (0.51 to 1.20) | <3 | ** | 1436 | 67.40 (56.44 to 80.48) |
| Day 8-14 | 59 | 1.09 (0.82 to 1.45) | 33 | 0.96 (0.65 to 1.42) | 10 | 3.33 (1.61 to 6.90) | 307 | 14.25 (11.55 to 17.57) |
| Day 15-21 | 56 | 1.17 (0.87 to 1.56) | 41 | 1.29 (0.89 to 1.87) | 5 | 1.61 (0.62 to 4.20) | 186 | 8.55 (6.77 to 10.81) |
| ***2^nd^ dose*** |  |  |  |  |  |  |  |  |
| Day 1-21 | 126 | 0.90 (0.71 to 1.13) | 85 | 0.85 (0.61 to 1.18) | 9 | 0.94 (0.39 to 2.28) | - | - |
| Day 1-7 | 41 | 0.86 (0.61 to 1.20) | 23 | 0.71 (0.44 to 1.14) | <3 | ** | - | - |
| Day 8-14 | 38 | 0.82 (0.58 to 1.16) | 28 | 0.85 (0.54 to 1.33) | 3 | 0.96 (0.27 to 3.45) | - | - |
| Day 15-21 | 47 | 1.04 (0.75 to 1.45) | 34 | 1.05 (0.69 to 1.61) | 4 | 1.34 (0.41 to 4.36) | - | - |

Control period include days from 1 February 2021 to 28 February 2022 before the exposure (first dose of COVID-19 vaccination or COVID-19 diagnosis), excluding the pre-risk period (-14 to -1 day before exposure), and day of exposure (day 0). Risk period was 1-21 days post exposure. Analysis adjusted for calendar months. Abbreviations: CI, confidence interval; IRR, incidence rate ratio. ** Value not computed for event count less than 3.

Appendix 5 Sensitivity analysis for self-controlled case series with exclusion of cases with any history of SARS-CoV-2 infection before and during observation period

|  |  | **BNT162b2** |  | **CoronaVac** |  | **ChAdOx1** |
| --- | --- | --- | --- | --- | --- | --- |
|  | **Event** | **IRR (95% CI)** | **Event** | **IRR (95% CI)** | **Event** | **IRR (95% CI)** |
| **Thrombocytopenia** |  |  |  |  |  |  |
| Control period | 1568 | 1.00 | 759 | 1.00 | 96 | 1.00 |
| ***1^st^ dose / infection*** |  |  |  |  |  |  |
| Day 1-21 | 90 | 0.82 (0.66 to 1.03) | 42 | 0.80 (0.57 to 1.13) | 10 | 1.51 (0.73 to 3.12) |
| Day 1-7 | 34 | 0.89 (0.63 to 1.26) | 11 | 0.61 (0.33 to 1.12) | 3 | 1.46 (0.44 to 4.86) |
| Day 8-14 | 36 | 0.95 (0.68 to 1.33) | 17 | 0.93 (0.56 to 1.53) | <3 | ** |
| Day 15-21 | 20 | 0.60 (0.39 to 0.94) | 14 | 0.87 (0.50 to 1.54) | 6 | 2.49 (1.03 to 6.04) |
| ***2^nd^ dose*** |  |  |  |  |  |  |
| Day 1-21 | 101 | 0.93 (0.76 to 1.15) | 52 | 0.97 (0.71 to 1.32) | 8 | 1.43 (0.64 to 3.20) |
| Day 1-7 | 32 | 0.88 (0.62 to 1.26) | 10 | 0.56 (0.30 to 1.06) | <3 | ** |
| Day 8-14 | 34 | 0.94 (0.67 to 1.33) | 23 | 1.29 (0.83 to 2.00) | 3 | 1.67 (0.50 to 5.58) |
| Day 15-21 | 35 | 0.97 (0.69 to 1.36) | 19 | 1.06 (0.66 to 1.71) | 3 | 1.43 (0.43 to 4.74) |
|  |  |  |  |  |  |  |
| **Venous thromboembolism** |  |  |  |  |  |  |
| Control period | 1772 | 1.00 | 865 | 1.00 | 128 | 1.00 |
| ***1^st^ dose / infection*** |  |  |  |  |  |  |
| Day 1-21 | 130 | 0.97 (0.81 to 1.17) | 84 | 1.06 (0.82 to 1.35) | 14 | 1.44 (0.77 to 2.67) |
| Day 1-7 | 37 | 0.80 (0.58 to 1.12) | 21 | 0.78 (0.50 to 1.23) | <3 | ** |
| Day 8-14 | 45 | 0.97 (0.72 to 1.31) | 26 | 0.94 (0.62 to 1.40) | 9 | 2.75 (1.32 to 5.75) |
| Day 15-21 | 48 | 1.17 (0.87 to 1.56) | 37 | 1.46 (1.04 to 2.07) | 4 | 1.18 (0.42 to 3.32) |
| ***2^nd^ dose*** |  |  |  |  |  |  |
| Day 1-21 | 108 | 0.84 (0.69 to 1.03) | 69 | 0.80 (0.61 to 1.04) | 7 | 0.53 (0.24 to 1.16) |
| Day 1-7 | 37 | 0.86 (0.62 to 1.20) | 19 | 0.68 (0.43 to 1.08) | <3 | ** |
| Day 8-14 | 28 | 0.65 (0.45 to 0.95) | 24 | 0.83 (0.55 to 1.26) | <3 | ** |
| Day 15-21 | 43 | 1.01 (0.74 to 1.37) | 26 | 0.89 (0.59 to 1.32) | 4 | 0.91 (0.33 to 2.53) |

Control period include days from 1 February 2021 to 28 February 2022 outside of the risk period (1-21 days post exposure), pre-risk period (-14 to -1 day before exposure), and day of exposure (day 0). Analysis adjusted for calendar months. Abbreviations: CI, confidence interval; IRR, incidence rate ratio. ** Value not computed for event count less than 3.

Appendix 6 SAFECOVAC study group members, non-author collaborators

| **Name** | **Institution** | **Role** |
| --- | --- | --- |
| Azuana, Ramli | National Pharmaceutical Regulatory Agency, Selangor, Malaysia | Co-investigator |
| Sing Chet, Lee | National Pharmaceutical Regulatory Agency, Selangor, Malaysia | Co-investigator |
| Sim Mei, Choo | National Pharmaceutical Regulatory Agency, Selangor, Malaysia | Co-investigator |
| Maheshwara Rao, Appanan | Disease Control Division, Ministry of Health, Putrajaya, Malaysia | Co-investigator |
| Teck Long, King | Clinical Research Centre, Sarawak General Hospital, Sarawak, Malaysia | Co-investigator |
| Chia How, Yen | Clinical Research Centre, Queen Elizabeth II Hospital, Sabah, Malaysia | Co-investigator |
| Emelyne Bani, Anak Jam | Clinical Research Centre, Queen Elizabeth II Hospital, Sabah, Malaysia | Co-investigator |
| Fatihah, Mahmud | Clinical Research Centre, Tengku Ampuan Afzan Hospital, Pahang, Malaysia | Co-investigator |
| Fariz Safhan, Mohamad Nor | Clinical Research Centre, Tengku Ampuan Afzan Hospital, Pahang, Malaysia | Co-investigator |
| Muhammad Hazrul, Badrul Hisham | Clinical Research Centre, Tengku Ampuan Afzan Hospital, Pahang, Malaysia | Co-investigator |
| Siti Nurhafizah, Saharudin | Clinical Research Centre, Shah Alam Hospital, Selangor, Malaysia | Co-investigator |
| Nor Aliya, Ayub | Clinical Research Centre, Kuala Lumpur Hospital, Kuala Lumpur, Malaysia | Co-investigator |
| Raj Kumar, Sevalingam | Clinical Research Centre, Kuala Lumpur Hospital, Kuala Lumpur, Malaysia | Co-investigator |
| Rashidah, Bahari | Clinical Research Centre, Putrajaya Hospital, Putrajaya, Malaysia | Co-investigator |
| Nor Nadziroh, Ibrahim | Clinical Research Centre, Putrajaya Hospital, Putrajaya, Malaysia | Co-investigator |
| Nurain, Mohd Noor | Clinical Research Centre, Putrajaya Hospital, Putrajaya, Malaysia | Co-investigator |
| Lisa, Mohamed Nor | Clinical Research Centre, Putrajaya Hospital, Putrajaya, Malaysia | Co-investigator |
| Nurul Huda, Zainal Abidin | Clinical Research Centre, Putrajaya Hospital, Putrajaya, Malaysia | Co-investigator |
| Hin Seng, Wong | Clinical Research Centre, Selayang Hospital, Selangor, Malaysia | Co-investigator |
| Siti Nur Akmal, Ghazali | Institute for Clinical Research, National Institutes of Health, Selangor, Malaysia | Research assistant |
| Nurul Afifah, Rozkhaidi | Institute for Clinical Research, National Institutes of Health, Selangor, Malaysia | Research assistant |
| Norzubaidatulhikmah, Shaduqi | Institute for Clinical Research, National Institutes of Health, Selangor, Malaysia | Research assistant |
| Hanisah, Hossain | Clinical Research Centre, Sarawak General Hospital, Sarawak, Malaysia | Research assistant |
| Jeannette Lay, Jieni | Clinical Research Centre, Queen Elizabeth II Hospital, Sabah, Malaysia | Research assistant |
| Nor Azizah, Mohamad Nazri | Institute for Clinical Research, National Institutes of Health, Selangor, Malaysia | Research assistant |
